# Exploring TEM Coherence Properties via Speckle Contrast Analysis in Coherent Electron Scattering of Amorphous Material

**DOI:** 10.3390/nano13233016

**Published:** 2023-11-24

**Authors:** Ji-Hwan Kwon, Joohyun Lee, Je In Lee, Byeong-Gwan Cho, Sooheyong Lee

**Affiliations:** 1 Korea Research Institute of Standards and Science, Daejeon 34113, Republic of Korea; kwonjh@kriss.re.kr (J.-H.K.); joohyun.lee@kriss.re.kr (J.L.); newbgcho@gmail.com (B.-G.C.); 2 Department of Nano Convergence Measurement, Korea University of Science and Technology, Daejeon 34113, Republic of Korea; 3 School of Materials Science and Engineering, Pusan National University, Busan 46241, Republic of Korea; jilee@pusan.ac.kr

**Keywords:** electron diffraction, speckle contrast, coherent diffraction, coherence, amorphous

## Abstract

We investigate the coherence properties of a transmission electron microscope by analyzing nano-diffraction speckles originating from bulk metallic glass. The spatial correlation function of the coherent diffraction patterns, obtained in the transmission geometry, reveals the highly coherent nature of the electron probe beam and its spatial dimension incident on the sample. Quantitative agreement between the measured speckle contrast and an analytical model yields estimates for the transverse and longitudinal coherence lengths of the source. We also demonstrate that the coherence can be controlled by changing the beam convergence angle. Our findings underscore the preservation of electron beam coherence throughout the electron optics, as evidenced by the high-contrast speckles observed in the scattering patterns of the amorphous system. This study paves the way for the application of advanced coherent diffraction methodologies to investigate local structures and dynamics occurring at atomic-length scales across a diverse range of materials.

## 1. Introduction

Modern transmission electron microscopes (TEM) utilize low-emittance sources to generate electron wavefields that deliver a well-collimated and transversely coherent electron beam [1]. This high degree of coherence coupled with short wavelength enables interference-driven measurements, such as high-resolution transmission electron microscopy (HR-TEM), lattice imaging, focal-series reconstruction of exit waves, and electron holography and diffractive imaging [2,3,4]. Despite the potential benefits, the scattering of coherent radiation from a medium also results in the formation of non-uniform, phase-shifted diffraction patterns, commonly known as speckles. The effect of the speckle phenomenon in electron scattering has been explored previously. In particular, there was a fluctuation electron microscopy experiment in which speckle intensity variance from thin amorphous carbon and silicon samples was measured and compared with the simulation results [5]. Their work shows that the details of beam interaction with the sample must be taken into account to obtain fully quantitative results.

In the visible regimes, the utilization of laser speckle statistics finds widespread application in characterizing material surface roughness through the correlation of coherent diffraction patterns [6,7]. The analysis of speckle intensity and time correlation also offers insights into the behavior of microscopic particles suspended in liquids or gels [8,9,10]. Nevertheless, the use of optical light is inherently confined by its limited wavelength, making it suitable for systems with length scales in the micron range or longer. In recent years, the arrival of advanced synchrotron sources has enabled experiments utilizing coherent X-rays to probe the nanoscale structures and dynamics of disordered systems such as nanocrystalline compacts, glasses, and amorphous materials. Coherent diffraction imaging [11] and temporal correlation measurements, such as X-ray Photon Correlation Spectroscopy (XPCS), have become valuable tools for studying the dynamics and structures of nanometer-sized colloids and polymers [12,13,14], as well as the fluctuations of ferromagnetic [15] and ferroelectric domain structures [16]. Moreover, speckle measurement and analysis are utilized to investigate the coherence properties of ultrafast X-ray sources, such as X-ray-free electron lasers (XFEL) [17,18]. Despite these advancements, significant challenges persist due to the weak scattering cross-section between X-rays and amorphous materials, which has limited their application to bulk material investigation.

Short-wavelength electron beams with high spatial coherence can effectively mitigate the challenges associated with scattering in amorphous systems by producing robust speckle patterns that reveal structures and heterogeneities at pertinent length scales. Thus, high-contrast electron speckles offer the opportunity to identify bond order in disordered structures [19,20,21] and track temporal dynamics via time-correlation spectroscopy [22]. These methods hold great potential for exploring low-dimensional systems, such as emerging quantum materials. However, the successful application of these methods requires a quantitative characterization of electron-beam coherence since the speckle patterns are closely tied to the coherence properties of the electron source. Despite several proposed methods based on Airy patterns analysis [23], electron holography [24], and interference fringe measurement in electron diffraction [25], measuring the coherence of high-energy electron beams remains a non-trivial task due to the small length scales involved.

In this work, we present measurements of coherent diffraction speckle patterns from amorphous materials. The coherence characteristics of the incoming electron beam are encoded in the statistics of the speckle patterns. By carefully analyzing the scattering intensity fluctuations at atomic length scales, we gain insights into both transverse and longitudinal coherence properties. Our methodology involves meticulously resolving distinct electron speckle patterns originating from bulk metallic glass (BMG), followed by an in-depth analysis of their contrast in relation to the momentum transfer, represented as $Q=\frac{4\pi }{\lambda }sin\left(\theta \right)$, where λ and θ are the electron wavelength and scattering angle, respectively. In our experimental configuration, we find that the speckle contrast carries negligible *Q* dependence, but it decreases as the convergence angle increases. This result indicates that the scattering volume of the electrons from the sample roughly matches the coherence volume, consequently influencing the speckle contrast. By modeling the response of the contrast relative to the incident beam size, we derive estimates for both the transverse and longitudinal coherence lengths of the TEM apparatus.

## 2. Materials and Methods

### 2.1. Sample Preparation

Zr${}_{46.0}$Cu${}_{30.1}$Ag${}_{8.4}$Al${}_{8.0}$Be${}_{7.5}$ bulk-metallic-glass-forming alloy was prepared by arc-melting a mixture of pure metals (Ag, Cu, and Zr, a purity of above 99.9%) and Cu${}_{77.3}$Be${}_{22.7}$ alloy under a Ti-gettered Ar atmosphere. The cylindrical BMG specimen with a diameter of 5 mm was obtained by suction casting into a copper mold. TEM specimen was prepared by mechanical polishing followed by dimple grinding and ion beam milling. The ion beam milling was performed using a Gatan Precision Ion Polishing System II for several hours at a voltage of 5.0 kV and milling angle of ±4°.

### 2.2. TEM Measurement

Electron diffraction was performed with the Spectra 300 (S)TEM equipped with the GIF Continuum K3 system installed at the Korea Research Institute of Standards and Science. The electron diffraction was completed in the microprobe mode. The size of the electron beam was fixed at approximately 14 nm, while the convergence angles on the sample were varied from 95 μrad to 360 μrad. The convergence angle (α) was changed by adjusting the strength of the C2 and C3 lenses in the microprobe mode, and we refer to the value of the convergence angle that is calibrated by the TEM manufacturer. To measure the coherent electron diffraction patterns in the counting mode, we used the K3 direct electron detector placed at the Gatan Imaging Filter (GIF) position. The TEM was operated at 300 kV and the energy spread of the electron beam was measured to be ∼1 eV, as determined by the zero-loss peak of the electron energy loss spectroscopy, resulting in ΔE/E ∼ 10${}^{-6}$. The beamstop was used to protect the direct electron detector from the strong intensity of the main transmitted electron beam. The electron diffraction was performed at a screen current of 30 pA, maintaining an average of less than 15 electrons/(pix·s) deposited on the detector, where the speckle contrast did not change for the duration of our experiment. Finally, we calibrated the diffraction pattern images using a gold crystal’s diffraction pattern as a reference. The acquired image consisted of 3456 × 3456 pixels, each pixel measuring 0.009 nm${}^{-1}$. The total accumulated dose employed in our experiment is approximately 2.0 $\times \;10^{4}$ e${}^{-}$/Å${}^{2}$, which is below the threshold known to cause damage to electron-beam-sensitive zeolites [26].

## 3. Results and Discussion

**Figure 1:**
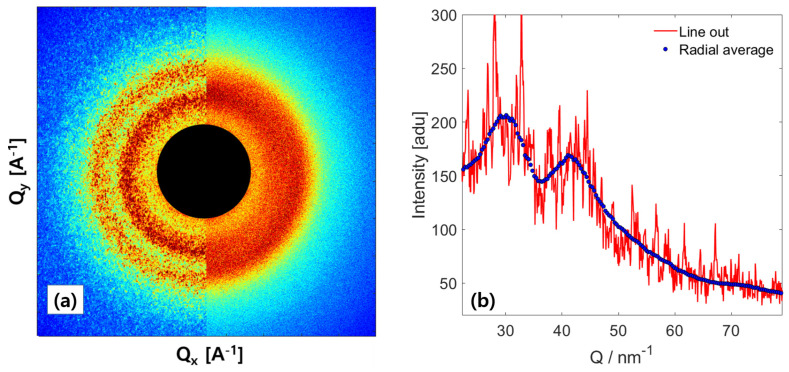
(**a**) The diffraction pattern from the bulk metallic glass exhibits coherent diffraction patterns with pronounced spatial intensity fluctuations (left half) when subjected to scattering with a well-collimated electron beam. In contrast, the diffraction pattern obtained from an amorphous sample using a highly converging beam displays a smooth concentric pattern (right half). (**b**) The intensity profile along the radial direction, as a function of *Q*, reveals significant fluctuations in intensity around the azimuthally averaged intensity I(Q).

Figure 1a presents the electron diffraction patterns acquired from the BMG sample using two different beam convergence parameters. In the left panel, we observe a nano-diffraction pattern captured by using the smallest convergence angle α = 95 μrad. The presence of grainy structures superimposed on the concentric ring (halo pattern) indicates well-developed speckles, reflecting the high coherence of the incident electron beam. On average, each detector pixel registers approximately 30 electrons, contributing to the observed speckle pattern. Conversely, the right panel of Figure 1a displays a diffraction pattern obtained with a significantly larger convergence angle of 380 μrad. The smooth structural rings observed here resemble typical diffraction peaks of a disordered system illuminated by an incoherent light source. In Figure 1b, the blue circles indicate the ensemble-averaged scattering $\langle I(Q,t)\rangle$, calculated through azimuthal averaging of the speckle pattern shown in Figure 1a (left panel). The diffuse and broad diffraction peaks clearly indicate the absence of long-range order and the presence of disordered atom distributions. The solid red line in Figure 1b represents a cut through the diffraction pattern from the beam center along the radial direction, where the intensity variations clearly exceed counting statistics. These speckle patterns observed in our study arise from the random arrangement of atoms within the material, with characteristic length scales of a few angstroms. While the intensity and positions of these diffraction peaks could offer insights into the local structure and spatial arrangement of atoms in the BMG sample, such details are beyond the scope of this study.

**Figure 2:**
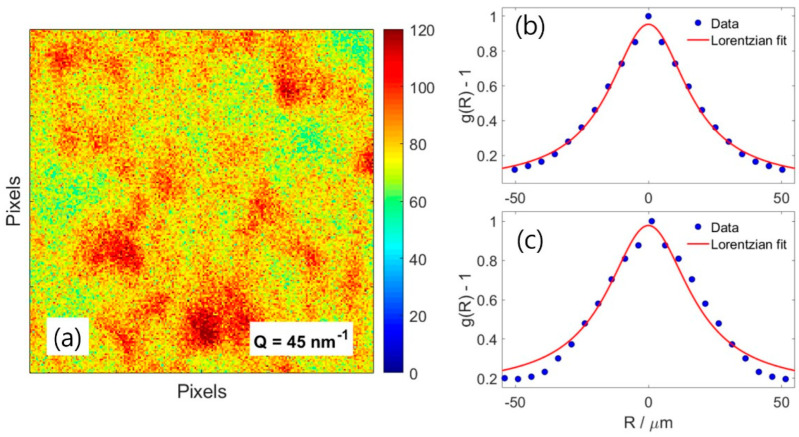
(**a**) Close-up view of the speckle pattern at *Q* = 45 nm${}^{-1}$. Spatial autocorrelation functions in horizontal (**b**) and vertical (**c**) directions yield the speckle widths along the two directions.

Figure 2a shows a close-up view of the scattering pattern near *Q* = 45 nm${}^{-1}$. The spatial intensity correlation function of the speckle pattern, denoted as g(R), offers insights into the mutual coherence function γ(R). The mutual coherence function along the horizontal and vertical directions, which are perpendicular to the incident beam, can be effectively modeled using an exponential relationship, $\gamma_{h,v}\left(R\right)=\exp\left(-R/R_{h,v}\right)$, where $R_{h,v}$ represents the speckle sizes along each direction [27]. Assuming a Lorentzian beam profile for both directions, we can establish a connection between the speckle size *R* and the electron beam size using the relation R=λL/d, where *L* stands for the sample-to-detector distance, and *d* is the electron beam size at the sample location. In our analysis, we explored the use of both Lorentzian and Gaussian distribution functions to fit the spatial intensity correlation function. Although fitting the data with both functions yielded comparable results, we chose to use the Lorentzian function. This choice was made because the intensity correlation functions shown in Figure 2b,c exhibit distributions with extended tails, and the Lorentzian function proved to be a better fit for these characteristics. Fitting the spatial intensity correlation function to the experimental data yields $R_{h}$ = 35.79 μm (see Figure 2b) and $R_{v}$ = 37.82 μm (see Figure 2c), from which we extract an electron beam size of 14.4 (h) × 13.6 (v) nm${}^{2}$ full-width half maximum (FWHM), which is consistent with the direct beam size measurement. This result demonstrates that, under our experimental conditions, the electron speckle is well-developed, and its features are effectively resolved by the 2D detector.

**Figure 3:**
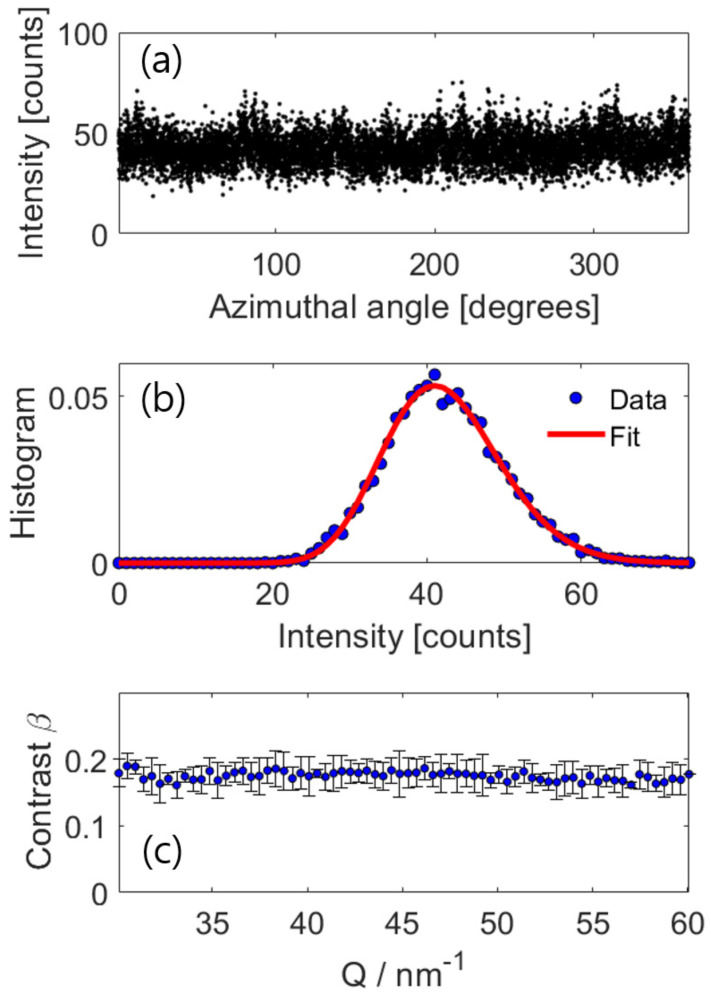
(**a**) Azimuthal intensity profile of scattered X-rays at *Q* = 60 nm${}^{-1}$ statistically distributed over 2π; (**b**) its histogram of the intensity distribution. Red solid line: fit according to Equation (1), from which the number of speckle modes is calculated; (**c**) speckle contrast β as a function of *Q*.

To perform the speckle contrast analysis, we define a region of interest in the diffraction pattern as an annulus with a radius ranging from *Q* = 30 to 60 nm${}^{-1}$ and a width of dQ = 0.027 nm${}^{-1}$ (approximately three pixels). Figure 3a shows the intensity fluctuation at *Q* = 60 nm${}^{-1}$ that is statistically distributed over 2π. When the illuminated sample volume is larger than the coherence volume, one can consider that the sample is composed of small *M* scattering sites that produce the intensity sum of *M* independent speckles patterns. Here, the scattered intensity *I* follows the gamma probability density distribution [28]

(1) $$P\left(I\right)=\frac{\Gamma (I+M)}{\Gamma (I+1)\Gamma (M)}{\left(1+\frac{M}{\langle I\rangle }\right)}^{-1}{\left(1+\frac{\langle I\rangle }{M}\right)}^{-M},$$

where *I* is the number of electron events and *M* is the number of speckle modes. Figure 3b displays the intensity histogram of the speckle pattern in the region of interest and the fitting result that is obtained by using Equation (1). Here, the speckle mode is determined by the ratio between the scattering and coherence volumes, and it provides the speckle contrast through relation $\beta =1/\sqrt{M}$ [17,18]. For instance, the intensity distribution shown in Figure 3b yields *M* = 31, which corresponds to a speckle contrast β = 0.18. The agreement between the data and the fit demonstrates that there is no scaled intensity below approximately 48% of the mean, implying the presence of a constant component within the incident beam that does not contribute to the formation of the coherent interference pattern. This incoherent fraction results in a reduction in measured contrast. This effect is likely attributed to the spatial coherence length being shorter than the size of the beam illuminating the sample surface. In previous electron microscopy studies, such incoherent contribution is referred to as contrast mismatch or “Stobbs factor” [29]. Its origin was attributed to the underestimation of the modulation transfer function (MTF) in the instrument [30] and the ensemble-averaged atomic motions occurring at a timescale faster than the detector response times [31,32]. As shown in Figure 3c, the speckle contrast fluctuates very closely to a mean value of β = 0.17 ± 0.02 throughout the whole *Q* range, indicating a partially coherent beam in the focal area of the electron beam in this experiment. The levels of speckle contrast shown in this study are consistent with previous work [33], in which normalized variance is investigated at varying convergence angles. However, for quantitative comparison with previous studies [23,33,34], further investigation needs to be carried out using the TEM under various experimental conditions.

## 4. Discussion

**Figure 4:**
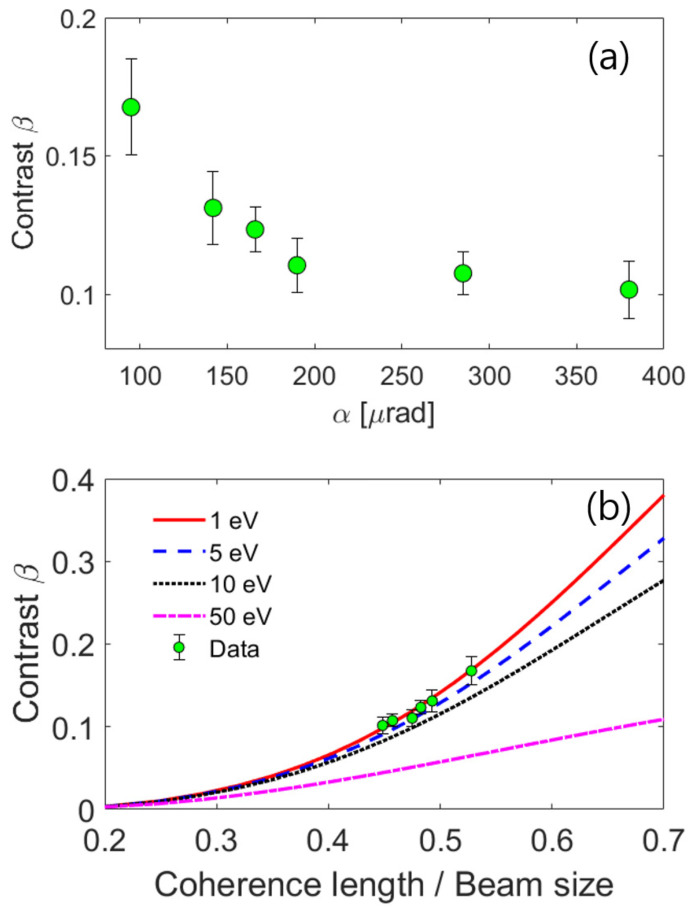
(**a**) The speckle contrast as a function of convergence angle; (**b**) comparison between experiment and calculation for different bandwidth parameters.

Coherence is composed of spatial and longitudinal components. Transverse coherence is a measure of the extent to which wavelength and the phase of a wave remain correlated, and its quality depends on how closely the source characteristics approach a point source. On the other hand, longitudinal coherence is related to the bandwidth Δλ/λ of the electron beam. In practice, these properties determine the quality of phase contrast images, the sharpness of electron diffraction patterns, and thus the quality of diffraction contrast images from crystalline materials. Although the coherence of a source is mostly governed by the instrument design, it can be affected by the operational parameters of the apparatus. For instance, operating TEM at higher energy or reducing its bandwidth by using a monochromator can significantly enhance its longitudinal coherence. In order to evaluate the coherence of the electron beam, we analyze the variations in speckle contrast at *Q* = 60 nm${}^{-1}$ as the convergence angle α in our instrument is varied from 95 to 360 μrad. As shown in Figure 4a, β initially decreases as the convergence angles increase. However, for the angles greater than α = 190 μrad, the contrast drop begins to stagnate. This inverse relation implies that the transverse coherence of the electron beam can be maximized by minimizing the α angle. In previous studies [35,36], it has been shown that the variation in the beam convergence angle has a notable effect on the coherence of the electron beam. We note that, even at the lowest α condition employed in this study, β is notably less than the ideal case of β = 1. These observations suggest that the coherent diffraction patterns encompass multiple speckles, indicating that the effective coherence volume produced by the electron beam is indeed smaller than the illuminated volume. We note that speckle contrast can be improved by increasing the coherence volume ratio, which can be achieved by optimizing the experimental conditions in the coherence diffraction measurement, such as reducing the beam size while preserving the convergence angle.

The coherence properties of radiation are reflected in the statistical properties of the speckle pattern. Nonetheless, the speckle contrast is not directly tied to beam coherence; instead, it is more closely associated with the ratio between the coherence volume and the scattering volume. To perform a more in-depth analysis of the speckle statistics, we refer to an analytical model, which has been successfully applied to characterize the coherence properties of XFEL sources. In the Fraunhofer regime, the relationship between the coherence of the incoming radiation and the resulting speckle contrast in the scattering patterns can be provided by [17]

(2) $$\beta =\frac{2}{L^{2}W^{2}}\int_{0}^{L}dx\left(L-x\right)\int_{0}^{W}dy\left(W-y\right)\exp\left(\frac{-x^{2}}{\xi^{2}}\right)\left|\exp\left(-\left|Ax+By\right|\right)+\exp\left(-\left|Ax-By\right|\right)\right|$$

with coefficients $A=\frac{\Delta \lambda }{\lambda }Q\sqrt{1-\frac{Q^{2}}{4k_{o}^{2}}}$ and $B=-\frac{\Delta \lambda }{2\lambda }\frac{Q^{2}}{k_{o}}$, where *L* represents the beam size, *W* is the sample thickness, and $k_{o}$ = 2$\frac{\pi }{\lambda }$ denotes the wavevector of the incoming electrons. Thus, the speckle contrast β is related to the degree of coherence of the incoming beam, where quantity is affected by both the bandwidth Δλ/λ and the spatial coherence ξ. Here, we performed the calculation with four different energy bandwidths ranging from 1 to 50 eV. We assess the degree of coherence for the electron beam by matching this analytical model to the experimental data (see Figure 4b). Our result yields estimates for transverse coherence length of 7.3 nm and longitudinal coherence of 416 nm, values that are consistent with those previously reported [37].

The ability to probe nanoscale phenomena requires a thorough understanding of how beam interactions with the sample [38], environment, and control parameters [39] affect the electron beam coherence properties. Recently, the delivery of ultrashort electron pulses, enabled by femtosecond-induced photoemission, allows probing structural dynamics occurring at nano- to femtosecond timescales. In such cases, the operating conditions of the optical laser, such as its spatial and temporal mode as well as its pulse structure, can have an impact on the coherence of the electron beam. It becomes crucial to assess its coherence properties on a pulse-to-pulse basis. Our results have applications in such cases.

## 5. Conclusions

We demonstrate measuring and quantifying electron speckle patterns that are generated from atomic-scale ordering within a bulk metallic glass using a transmission electron microscope operating at 300 kV acceleration voltage. The high-contrast factors in electron speckle patterns indicate that the electron beam carries a high degree of coherence, which is sufficient to perform coherent diffraction measurements and analysis to study atomic-scale structures and dynamics. Moreover, employing a simple analytical model, we have effectively determined both the transverse and longitudinal coherence lengths of the electron beam. We expect that this result will be affected by different acceleration voltages. Specifically, given that the energy bandwidth remains unchanged, increasing the acceleration voltage should lead to an increase in the longitudinal coherence, whereas an opposite effect is expected at lower energies. Conversely, we expect that its influence on spatial coherence will not be as significant. This observation suggests that operating the TEM at a higher acceleration voltage would be more advantageous for achieving speckles with heightened contrast. This research not only enhances our understanding of these coherence characteristics but also serves as a blueprint for designing more intricate experiments that harness speckle pattern analysis to extract atomic-scale information.

## Data Availability

The data presented in this study are available on request from the corresponding author.
